# Case Report: Combination of focal vibration therapy and botulinum toxin injections to treat equinus gait in a child with unilateral spastic cerebral palsy

**DOI:** 10.3389/fresc.2025.1454109

**Published:** 2025-02-06

**Authors:** Christophe Boulay, Jacques-Olivier Coq, Morgan Sangeux, Guillaume Authier, Alexis Ulian, Maud Pradines, Marjolaine Baude, Béatrice Desnous, Jean-Luc Jouve, Bernard Parratte, Emilie Peltier, Sébastien Pesenti, Jean-Michel Gracies

**Affiliations:** ^1^ Gait Laboratory, Pediatric Orthopaedic Surgery Department, Timone Children’s Hospital, Marseille, France; ^2^ Institut des Sciences du Mouvement (ISM), Equipe DynamiCC, UMR 7287 CNRS/Aix Marseille Université, Marseille, France; ^3^ AP-HP, Service de Rééducation Neurolocomotrice, Unité de Neurorééducation, Hôpitaux Universitaires Henri Mondor, Créteil, France; ^4^ Department of Orthopaedics, University Children’s Hospital, Basel, Switzerland; ^5^ UR 7377 BIOTN, Laboratoire Analyse et Restauration du Mouvement, Université Paris Est Créteil (UPEC), Créteil, France; ^6^ Pediatric Neurology Department, Timone Children’s Hospital, Marseille, France

**Keywords:** children with cerebral palsy, equinus, focal vibration therapy, botulinum neurotoxin A, five-step assessment

## Abstract

**Introduction:**

Focal vibration therapy (FVT) is increasingly used in the treatment of spastic paresis. In adults, it has been shown to reduce spasticity and to increase torque production from the vibrated muscles by restoring reciprocal inhibition of antagonists, thereby improving overall gait. In children with spastic cerebral palsy (CP), FVT has also been suggested to reduce spasticity, increase torque production and improve gait function, but evidence is limited.

**Methods:**

We report the case of a child with unilateral spastic CP (USCP) and equinus gait (GFMCS II level) with (i) ankle dorsiflexor paresis, (ii) ankle plantar flexor overactivity, especially in gastrosoleus complex and peroneus longus, (iii) spastic myopathy, affecting gastrosoleus complex in particular, and (iv) calf pain seemingly related to muscle overactivity. The child was treated with a two-month program of alternating dorsiflexor and plantar flexor focal vibration therapy (FVT) and botulinum neurotoxin A (BoNT-A) injections into plantar flexors, alongside conventional physiotherapy.

**Results and discussion:**

Clinical evaluations during the two-month program showed (i) improved walking speed (ii) decreased ankle dorsiflexor paresis and ankle plantar flexor overactivity, especially spastic co-contraction and spasticity, (iii) improved passive extensibility in plantar flexors, and (iv) reduced pain. This is the first report of the combination of FVT and BoNT-A injections having promising effects on equinus gait in USCP.

## 1 Introduction

Cerebral palsy (CP) is a developmental motor disorder affecting about 2.5 children per 1000 live births in developed countries (1), caused by non-progressive brain injury before or shortly after birth, with reduced descending drive that secondarily hinders spinal cord development (2). CP manifests as both a neurological disorder (antagonist overactivity and agonist paresis) and a muscle disorder (spastic myopathy with stiffening of antagonists) (3, 4). At the ankle, there are three main forms of plantar flexor overactivity in children with CP: (i) *spastic cocontraction*, defined as unwanted, involuntary plantar flexor activation during voluntary dorsiflexor effort, primarily due to misdirection of the supraspinal descending drive, leading to reduced ankle dorsiflexion range of motion (3–6); (ii) *spastic dystonia*, namely unwanted, involuntary muscle activation at rest, in the absence of stretching or voluntary effort (3, 7), and (iii) *spasticity*, defined as increased velocity-dependent responses to phasic stretch, detected and measured at rest (3, 8). Children with CP also present with dorsiflexor paresis that is worsened by plantar flexor stretch, known as *stretch-sensitive paresis* (3, 4, 9).

Two vicious cycles ensue in that context: agonist paresis both causes and becomes worsened by disuse, and spastic myopathy both causes and becomes worsened by overactivity (8, 10). Musculoskeletal deformities acquired during growth have been classically handled by tenotomy or fascia- and/or aponeurosis of muscle(s) release surgery but the underlying spastic myopathy and misdirection of the supraspinal drive remain. Yet, early management, long before growth spurts, is crucial to minimize the emergence of deformities. There is early evidence that focal vibration therapy (FVT) and botulinum neurotoxin A (BoNT-A) injections may improve patient outcomes in this context (11–13).

Repeated sessions of focal vibrations (12) generated with a mechanical or pneumatic device have been reported to reduce resistance to passive movement from the vibrated plantar flexors in paretic patients (14) and to increase voluntary torque production from vibrated dorsiflexors in healthy subjects (15). Furthermore, gait function has been reported to be improved by FVT applied to dorsiflexors in adults after stroke (16), and to plantar flexors in children with CP (12, 17). Vibrations of aponeurosis-tendon junctions activate spindles, inducing primarily Ia excitation, which increases excitability in the corticospinal pathway to the vibrated muscle (17–20) and potentially also to the non-vibrated muscle (18). FVT may therefore be beneficial in children with CP; its safety and efficacy have been suggested in children both for the neuromuscular manifestations of CP and to treat CP-related pain (16, 21–32).

The effects of FVT following BoNT-A injections is unknown but *in vitro* and clinical data (33, 34) suggest that the increase in muscle temperature and activation induced by FVT may enhance the translocation of the BoNT-A light chain into the presynaptic terminal and thus increase toxin potency and duration in the vibrated muscle (33, 34). Here, we present the case of a child with CP who was treated with plantar flexor FVT before and after BoNT-A injections. The child was evaluated throughout the treatment program using the Five-Step Assessment (FSA) (35), which quantifies the extent of the various components of deforming spastic paresis.

## 2 Case description

**Figure 1:**
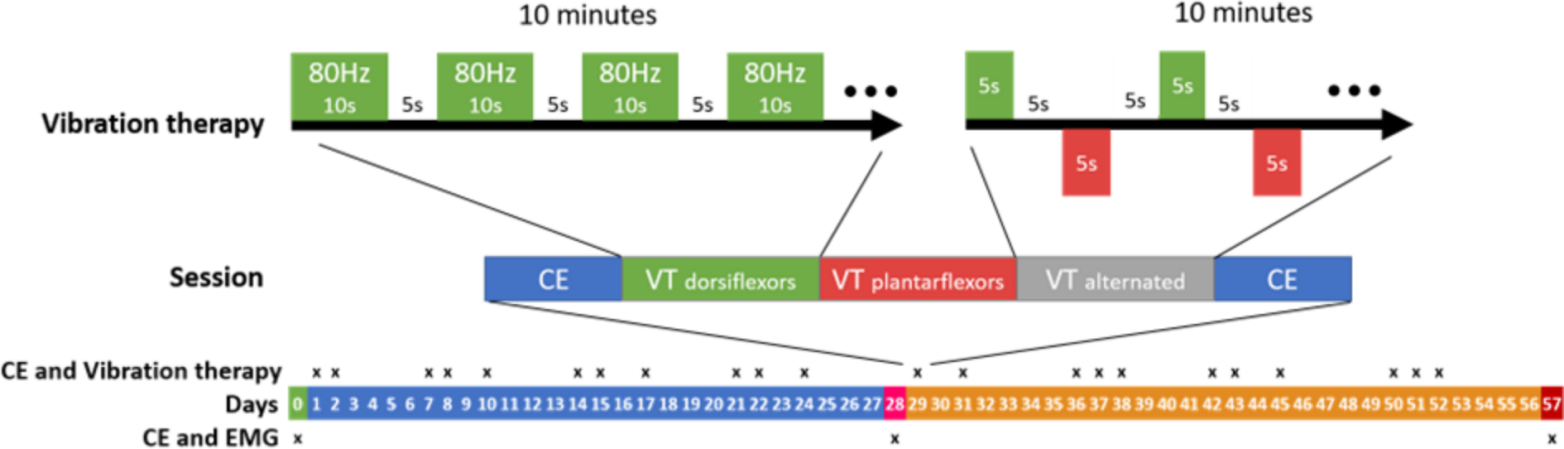
Details of the two-month treatment program based on focal vibration therapy (FVT)and botulinum neurotoxin A injections. The injections were performed on day 28. Each FVT session involved 10 min of vibration blocks (80 Hz vibration for 10 s every 5 s) applied to dorsiflexors followed by 10 min of the same vibration blocks applied to the plantar flexors, followed by 10 min of alternate vibration blocks applied to dorsiflexors and plantar flexors (80 Hz for 5 s every 5 s). Each session started and ended with a clinical examination (CE), including patient-assessment of illusory movements triggered by applying vibrations to agonist or antagonist muscles.

The child, a 5-year-old boy (18.5 kg, 1.09 m) with confirmed diagnosis of unilateral spastic cerebral palsy (USCP) and gross motor functional classification (GMFCS) II, was followed longitudinally for one month of FVT applied to dorsi- and plantar flexor muscles (Vibrasens, Techno Concept, serial number 14-V.01-210, Manosque, France) before and after plantar flexor injections of BoNT-A (Figure 1). During FVT sessions, vibrations were applied alternatingly to the myotendinous junction of the gastrosoleus complex (GSC) and tibialis anterior (TA; see details in Figure 1). On D28, the patient received a total of 15 U/kg abobotulinumtoxin A in gastrocnemius medialis (GM, 60 U), gastrocnemius lateralis (GL, 30 U), peroneus longus (PL, 90 U) and soleus (SOL, 90 U). Injections were performed under local anesthesia with ultrasound guidance.

## 3 Diagnostic assessment

Clinical assessments, including quantified clinical examination (FSA), electromyography (EMG, data to be reported elsewhere) during walking, and walking speed measurements, were performed before FVT (D0), the morning of the BoNT-A injections (D28), and 29 days after the BoNT-A injections (D57). The patient received 11 FVT sessions between D0 and D28, and another 11 sessions between D28 and D57, as shown in Figure 1. Clinical examinations consisting of FSA measurements and quantified child-perception of illusory movements (20, 36), were performed at the beginning and end of each FVT session to evaluate immediate effects. Conventional physical therapy program for equinus gait continued unchanged during the two-month FVT program. This included plantar flexor stretching and strengthening of command on TA and on plantar flexor muscles once or twice a week.

### 3.1 Five-step assessment

FSA measurements (first three steps of the FSA) were performed using 0° defined as the position of minimum stretch of the tested muscle (35). Ankle joint range of dorsiflexion measurements against the resistance of the soleus (knee flexed) and of the GSC (knee extended) were performed with the patient in supine position. The range of motion of the ankle was measured between the fibula and the posterior half of the external border of the foot, to cancel effects of any foot pronation during dorsiflexion efforts. Maximum clinical extensibility was estimated by X_V1_, the *angle of arrest* under slow stretching. Stretch reflex threshold was quantified by X_V3_, the *angle of catch* or clonus under brisk passive stretching. The *angle of match* between maximal voluntary activation of dorsiflexors and passive and active resistance of the plantar flexors was quantified by X_A_, i.e., the range of ankle dorsiflexion reached during maximum voluntary ankle dorsiflexion efforts, in the knee extended and knee flexed positions.

### 3.2 Illusory movements

Illusory movements related to vibration were self-assessed by the child immediately before and after each FVT session (36, 37). Illusory movements were classified as antagonist vibratory response (AVR, the physiological response) if vibrations applied to the myotendinous junction triggered illusory movement in the opposite direction, i.e., felt by the brain as *stretch* of the vibrated muscle, or tonic vibratory response (TVR) if vibrations applied to the myotendinous junction triggered illusory movement corresponding to the perception of *contraction* of the vibrated muscle. Results in either direction were reported on an ordinal scale from 0–3, with 0, 1, 2 and 3 indicating no sensation of movement, less than 5° of sensed movement, 5–10° of sensed movement, and more than 10° of sensed movement, respectively.

### 3.3 Statistics

Mean differences between pre- and post-session FSA variables, before and after BoNT-A injections, were summarized as median (range), and tested for significance using paired *t*-tests or Wilcoxon tests depending on the conditions. Overall trends in the FSA variables as a function of time before and after BoNT-A injections were fitted using linear regression models and tested for significance using *t*-tests. Results were summarized as linear regression coefficients with 95% confidence intervals. All analyses were performed in R (version 4.3.2) (38). Statistical significance was defined as *p* < 0.05.

## 4 Results

**Figure 2:**
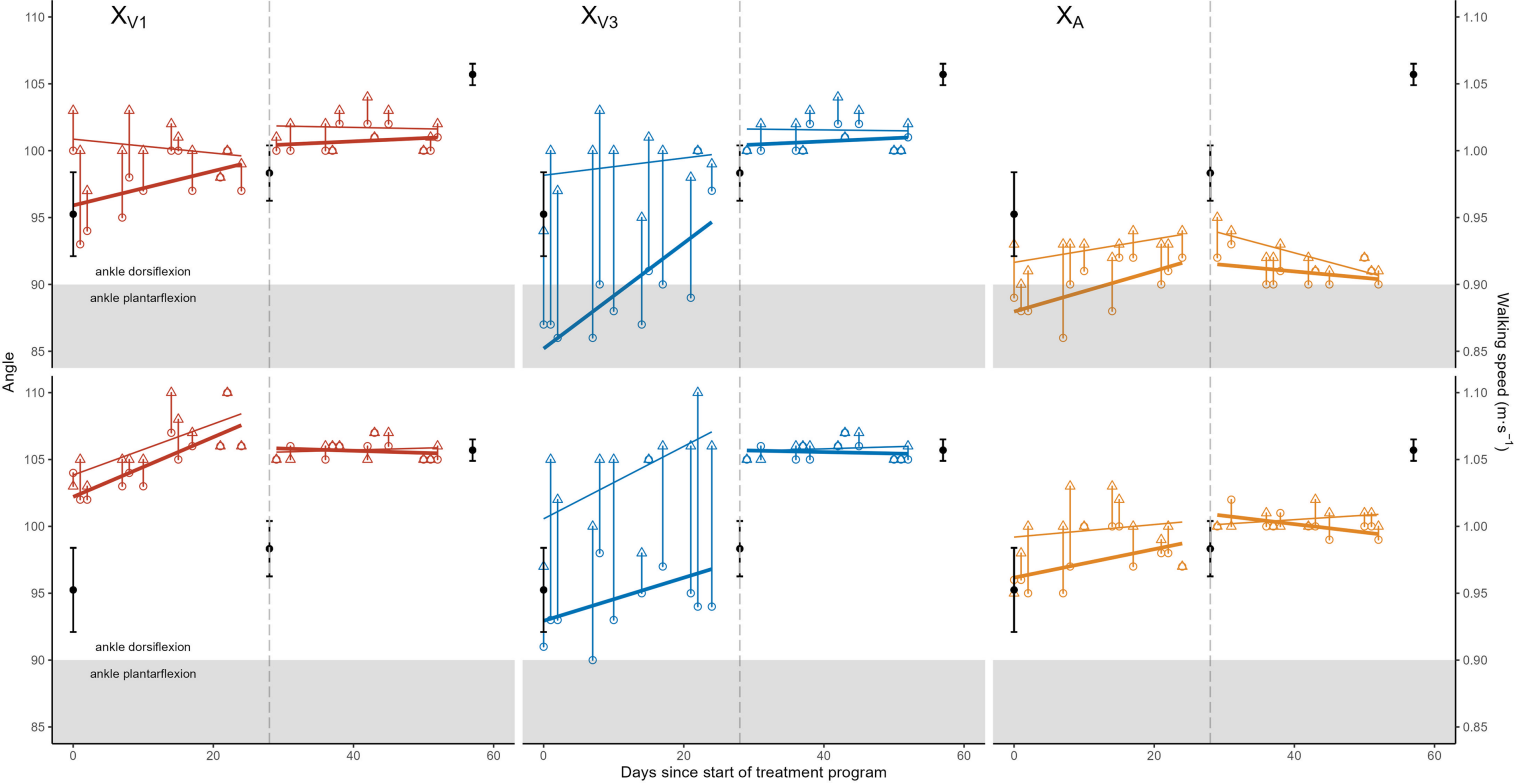
Range of motion (angle in degrees) as a function of time for the Five Step Assessment variables XV1 (red, left), XV3 (blue, middle) and XA (gold, right) in knee extended (top row) and knee extended (bottom row) positions. Pre-focal vibration therapy (FVT) session data are shown as open circles and post-FVT session data are shown as open triangles, with a vertical line linking pre- and post-session values from the same session. Separate lines of best fit are shown through the pre- and post-session values, both before and after botulinum neurotoxin A injections (represented by a vertical dashed line). Walking speeds (secondary *y*-axis on the right) are shown as solid black points with error bars in all panels.

Before BoNT-A injections, all three FSA variables (X_V1_, X_V3_, and X_A_) increased significantly during FVT sessions both in the knee flexed and knee extended positions (Table 1, Figure 2), with average gains of 1–3° for X_V1_ and X_A_ and 9° for X_V3_. After BoNT-A injections, the increases in X_V1_, X_V3_, and X_A_ between the start and end of each FVT session virtually disappeared, remaining only to a small extent in the knee extended position (Table 1, Figure 2).

**Table 1:** Mean differences between post- and pre-session values of Five Step Assessment (FSA) variables, before and after botulinum neurotoxin A (BoNT-A) injection.

	Before BoNT–A injection		After BoNT–A injection	
FSA Variables	Mean difference (after—before session)	*p* ^a^	Mean difference (after—before session)	*p* ^a^
Knee flexed				
X_V1_	1.25 [0.39; 2.11]	0.009	0.09 [–0.38; 0.56]	0.676
X_V3_	8.92 [6.13; 11.7]	<.001	0.27 [–0.16; 0.71]	0.192
X_A_	2.33 [0.94; 3.72]	0.004	0.45 [–0.36; 1.27]	0.242
Knee extended				
X_V1_	2.83 [1.51; 4.16]	0.001	1 [0.48; 1.52]	0.002
X_V3_	9.08 [6.33; 11.84]	<.001	0.82 [0.23; 1.41]	0.011
X_A_	2.92 [1.92; 3.91]	<.001	1.27 [0.59; 1.95]	0.002

Results are reported as best estimate [95% confidence interval].

X_V1_, angle of arrest under slow stretching; X_V3_, angle of catch/clonus under brisk passive stretching; X_A_, range of ankle dorsiflexion reached during maximum voluntary ankle dorsiflexion efforts.

^a^ Paired *t*-test.

Overall, Figure 2 shows the cumulative effects of the FVT program that included a general improvement (increase) in FSA variables before BoNT-A injections, up to level that remained approximately constant after BoNT-A injections. The pre-injection increases were significant for pre- and post-session X_V1_ values in the knee flexed position, post-session X_V3_ values in the knee flexed position, pre-session X_V3_ values in the knee extended position, and pre- and post-session X_A_ values in the knee extended position (Table 2). After BoNT-A injections, the only statistically significant variations were small decreases in pre-session X_A_ values in the knee flexed position and in post-session X_A_ values in the knee extended position (Table 2).

**Table 2:** Linear regression coefficients for pre- and post-session values of FSA variables as a function of time before and after botulinum neurotoxin A (BoNT-A) injection.

Variable	Before BoNT−A injection		After BoNT−A injection	
	Regression coefficient	*p* ^a^	Regression coefficient	*p* ^a^
Knee flexed				
X_V1_ pre	0.22 [0.11; 0.34]	0.002	−0.02 [−0.08; 0.05]	0.581
X_V1_ post	0.19 [0.05; 0.33]	0.013	0.01 [−0.06; 0.09]	0.675
X_V3_ pre	0.16 [−0.15; 0.47]	0.271	−0.01 [−0.08; 0.05]	0.719
X_V3_ post	0.27 [0.02; 0.52]	0.038	0.02 [−0.06; 0.09]	0.634
X_A_ pre	0.11 [−0.03; 0.24]	0.108	−0.06 [−0.13; 0]	0.058
X_A_ post	0.05 [−0.15; 0.24]	0.602	0.03 [−0.03; 0.09]	0.253
Knee extended				
X_V1_ pre	0.13 [−0.05; 0.31]	0.149	0.02 [−0.06; 0.11]	0.538
X_V1_ post	−0.05 [−0.2; 0.1]	0.451	−0.01 [−0.13; 0.11]	0.857
X_V3_ pre	0.39 [0.15; 0.64]	0.005	0.02 [−0.06; 0.11]	0.538
X_V3_ post	0.06 [−0.14; 0.27]	0.507	−0.01 [−0.14; 0.13]	0.925
X_A_ pre	0.15 [0.03; 0.28]	0.022	−0.05 [−0.14; 0.05]	0.273
X_A_ post	0.09 [0.01; 0.16]	0.028	−0.14 [−0.21; −0.07]	0.002

Results are reported as best estimate [95% confidence interval].

X_V1_, angle of arrest under slow stretching; X_V3_, angle of catch/clonus under brisk passive stretching; X_A_, range of ankle dorsiflexion reached during maximum voluntary ankle dorsiflexion efforts.

^a^ Linear regression *t*-test.

Between the beginning and the end of the two-month treatment program (comparing the mean of the first three pre-session values to the mean of the last-three post-session values), the overall angular gains were +2.7° [0.6; 4.7] for X_V1_, +13.0° [10.9; 15.1] for X_V3_ and +5.0° [3.7; 6.3] for X_A_, knee flexed, and +5.3° [−0.9; 11.6] for X_V1_, +14.0° [11.9; 16.1]° for X_V3_ and +3.0° [1.7; 4.3] for X_A_, knee extended. There was an associated +0.11 m/sec (+12%) increase in walking speed from 0.95 ± 0.06 m/sec (mean ± standard error) at the start of the treatment program to 1.06 ± 0.02 m/sec at the end of the program, which was more marked in the second half of the program, after BoNT-A injection (Figure 2). As the treatment program progressed, the child and his parents reported gradual reduction of calf pain.

Illusory movements before BoNT-A injections indicated normal AVR [median level of perceived plantar flexion movement, 3/3; interquartile range (IQR), 0, both before and after FVT sessions] when vibrations were applied to the tibialis anterior (agonist), while vibrations applied to the gastrocsoleus complex (antagonists) triggered no response (median level of perceived plantar flexion movement, 0/3; IQR, 0, both before and after FVT sessions). After BoNT-A injections (the following morning), the child sensed normal AVR when vibrations were applied to the gastrosoleus complex (median, 3/3; IQR, 1.5 before FVT sessions and median 2/3; IQR, 1 after FVT sessions). After these BoNT-A injections into plantar flexors, the child continued to report normal AVR-type illusory movements upon vibration of the tibialis anterior (median, 3/3; IQR, 1, both before and after FVT sessions).

## 5 Discussion

In this 5-year-old child with USCP, a treatment program comprising 22 alternating dorsi- and plantar flexor FVT sessions over two months with BoNT-A injections in the calf muscles (peroneus longus, soleus, gastrocnemii medialis and lateralis) (39) after one month, alongside continued conventional physiotherapy, was associated with improved walking speed and passive and active dorsiflexion movements and reduced calf pain.

Our results show that FVT sessions had immediate (session-to-session) and longer-term compound effects on clinical variables: increased maximal passive extensibility of the gastrocnemius muscle (increase in X_V1_), reduced spasticity (increase in X_V3_), reduced ankle dorsiflexor paresis or reduced passive and active resistance from plantar flexors (increase in X_A_). In this patient, FVT thus provided significant therapeutic benefits over conventional physiotherapy alone and our results support previous findings that FVT on agonists reduces spasticity in antagonists, perhaps by restoring pre-synaptic inhibition on Ia afferents, and increases agonist torque production, perhaps by increasing the excitability of corticospinal pathways to the vibrated agonists, thereby allowing better regulation between agonists and antagonists (40, 41).

An interesting finding was the quasi-disappearance of the FVT effects following BoNT-A injections into plantar flexors. This may relate to the gamma motoneuron and thus intrafusal blocking due to BoNT-A (42–44), thereby confirming that FVT effects may indeed use spindles as primary medium. The slight increases pursued in the knee extended position highlight the potential benefits of continued FVT in this position. The overall gains achieved for this child over the course of the two-month treatment program compare favorably with those reported by Camerota et al. (21) in a 5-year-old child with CP, after one month of FVT applied directly to the GSC alone (rather than repeated alternate applications to the myotendinous junction of the GSC and TA here).

BoNT-A injections also seemed to have a remarkable effect on patient-assessed illusory movements in this child, who perceived no illusory movement (“TVR-type” response) when vibrations were applied to the gastrocsoleus complex prior to injections, but felt between 5 and 10° of illusory movements into dorsiflexion (the physiological AVR response) after plantar flexor injections. These results may suggest that gastrosoleus complex may have become slightly more extensible (at least in the knee extended position) after injection, with mechanical stretching effects becoming more detectable by the child's brain after injections.

A limitation in this case report is that the post BoNT-A effects were not followed up for a full three months post injection, which might have precluded the evaluation of the full functional effects of the injection (45). In summary, this case highlights the potential benefits of combined FVT, BoNT-A injections and conventional physiotherapy in children with UCSP. Larger studies are required to confirm these preliminary findings.

## Data Availability

The raw data supporting the conclusions of this article will be made available by the authors, without undue reservation.
